# Intestinal tuberculosis masquerading as difficult to treat Crohn disease: a case report

**DOI:** 10.1186/s13104-016-2222-0

**Published:** 2016-08-24

**Authors:** Madunil A. Niriella, S. Kuleesha Kodisinghe, Arjuna P. De Silva, Janaki Hewavisenthi, Hithanadura J. de Silva

**Affiliations:** 1 Department of Medicine, Faculty of Medicine, University of Kelaniya, P O Box 6, Thalagolla Road, Ragama, GQ 11010 Sri Lanka; 2 University Medical Unit, Colombo North Teaching Hospital, Ragama, Sri Lanka

**Keywords:** Intestinal, Crohn disease, Tuberculosis

## Abstract

**Background:**

Crohn disease has low prevalence in Sri Lanka while compared to the West, while intestinal tuberculosis is common in the region. Since clinical, endoscopic and investigation features of Crohn disease overlap with intestinal tuberculosis, differentiating these two conditions becomes a dilemma for the clinician in the intestinal tuberculosis endemic setting.

**Case summary:**

An 18-year old Sri Lankan Muslim female presented with chronic abdominal pain and weight loss. Colonoscopy revealed an ulcerated ileocaecal valve and a terminal ileal stricture. Biopsy confirmed Crohn disease with no supportive features to suggest intestinal tuberculosis. Despite treatment with adequate immunosuppression she failed to improve and underwent a limited right hemicolectomy and terminal ileal resection. Histology confirmed intestinal tuberculosis and she made full recover with 6 months of anti-tuberculosis treatment.

**Conclusion:**

This case illustrates the importance of reviewing the diagnosis to include intestinal tuberculosis in an endemic setting, when already diagnosed Crohn disease is treatment refractory.

## Background

The prevalence of Crohn disease (CD) is low in Sri Lanka compared to the West. However, Crohn disease is increasingly diagnosed in recent years [1]. Intestinal tuberculosis (ITB) is common in the South Asian region, but the exact prevalence is not reported from Sri Lanka. There is overlap of clinical, endoscopic and investigation features of CD and ITB. Therefore, differentiating these two conditions is a challenge for the clinician, especially among patients unresponsive to initial treatment (Table 1). Furthermore, treatment as for CD in the setting of missed ITB, especially with immunosuppresses and biologics, can be life threatening.

**Table 1:** Clinical, endoscopic and radiological features which aid in differentiation of Crohn disease from intestinal tuberculosis [2, 3]

Crohn disease	Intestinal tuberculosis
*Clinical*	
Prolonged remitting and relapsing course	Continuous disease of short duration
Bleeding per rectum	High fever
Diarrhoea	Ascites
Perianal disease	
Intestinal fistulae	
Extra-intestinal manifestations	
*Endoscopic*	
Longitudinal ulcers	Transverse ulcers
Aphthous ulcers	Ulcer scars
Cobblestone-like mucosa	Patulous ileocaecal valve
Isolated terminal ileal involvement with relative caecal sparing	
Anorectal lesions	
*Contrast enhanced CT*	
Multiple levels of involvement	Involvement of less than four segments
Symmetric and concentric bowel wall thickening	Asymmetric bowel wall thickening
Mural stratification (target sign)	Large necrotic mesenteric lymph nodes
Increased mesenteric vascular stranding (comb sign)	
Fibrofatty proliferation in the mesentery (pathognomonic)	

## Case presentation

An 18 year old Sri Lankan Muslim female, presented with a 9 month history of colicky right iliac fossa pain and a 5 kg weight loss. She did not have diarrhoea, extra-intestinal manifestations of CD, and contact or past history of tuberculosis (TB). Clinical examination was normal.

Colonoscopy revealed an ulcerated ileocaecal valve and a terminal ileal stricture, while the colon itself was macroscopically normal. Biopsy of these lesions was reported as suggestive of CD. TB polymerase chain reaction (PCR) performed on the specimen was negative. Contrast-enhanced CT abdomen showed a segment of terminal ileum with concentric wall thickening. Mantoux test and Quantiferon gold test for TB were negative and chest X-ray was normal.

She was started on treatment for CD with a short course of oral steroids followed by azathioprine. However, she failed to tolerate azathioprine and subsequently 6-mercaptopurine (6-MP), and therefore was started on intravenous infliximab. Despite 3 dose of infliximab she failed to respond, and a follow up colonoscopy showed persistence of the ileal stricture. Therefore she was referred for surgical resection of the involved ileal segment. During the laparotomy, multiple terminal ileal and cecal strictures were noted and unexpectedly, multiple omental and peritoneal deposits were also seen. A limited right hemicolectomy including resection of the involved terminal ileum was performed.

**Fig. 1:**
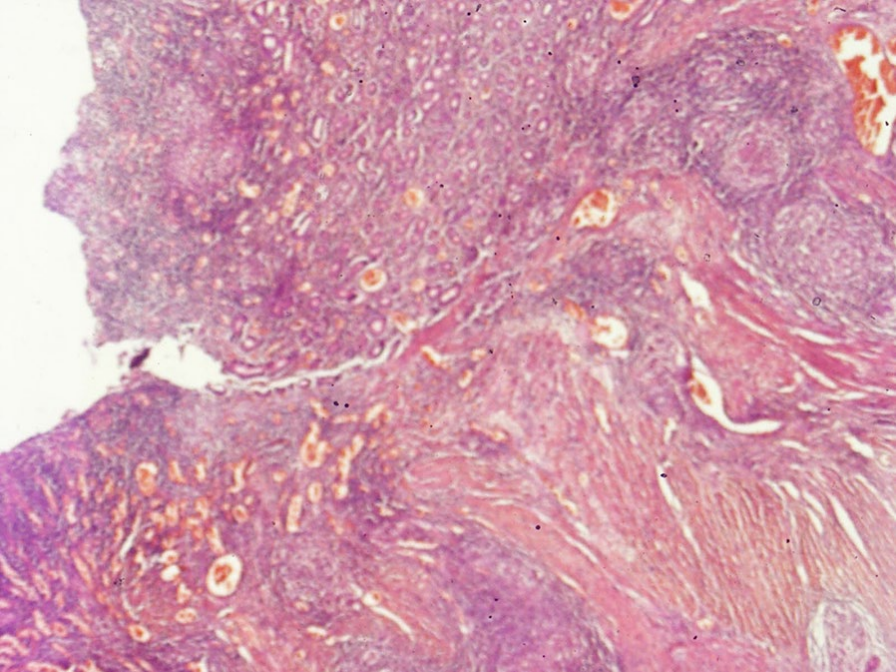
Confluent granulomata within the muscularis propria with ulceration of the overlying colonic mucosa. H & E ×40

**Fig. 2:**
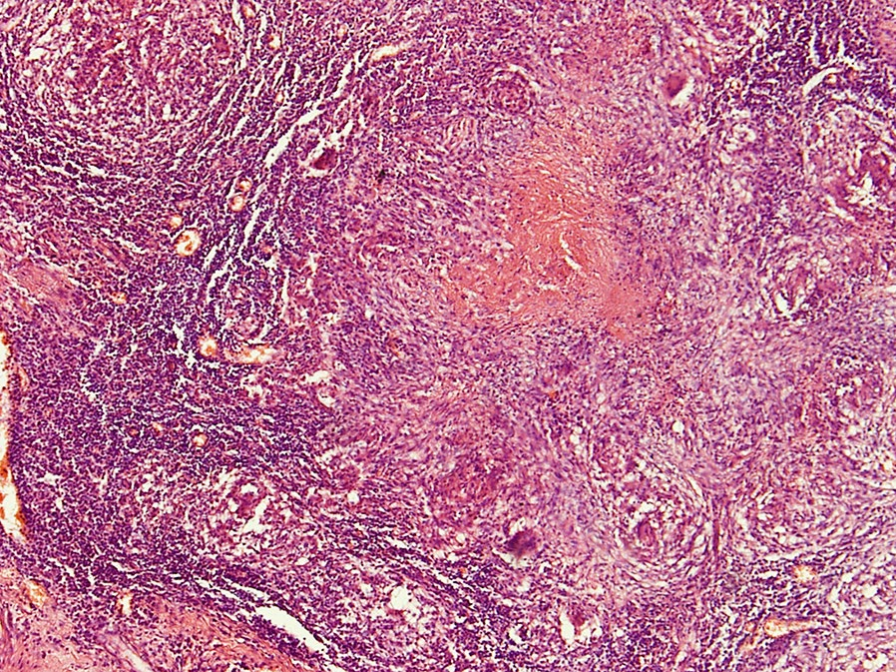
Confluent granulomata with central caseation H & E ×100

Histology of the resected specimen showed multiple coalescent caseating granulomata involving full thickness of the bowel wall and extending into the mesentery and pericolic fat, suggesting a diagnosis of ITB (Figs. 1, 2). No evidence of dissemination beyond the intestine was found. She was started on anti-TB treatment (ATT), and 2 months later was free of abdominal pain and has regained 3 kg of weight. Three months after commencing ATT a repeat colonoscopy was performed. There were no macroscopic or microscopic changes in the neo-terminal ileum or residual colon. She completed 6 months of ATT and made a full recovery.

## Discussion

The possibilities here are that the patient had ITB from the onset which was misdiagnosed as CD, or that the patient contracted ITB during immunosuppression with infliximab. There are numerous case reports in the literature where ITB was misdiagnosed as CD. There are also many reports of patients on infliximab developing TB, but none of these patients had TB isolated to the intestine. The most likely explanation in this patient is that of ITB misdiagnosed as CD. This is given the possibility of false negative skin test and TB PCR at initial evaluation, poor response to successive immunosuppression therapy, further worsening of bowel symptoms with infliximab therapy needing surgery and the complete resolution of clinical and endoscopic features with a course of ATT.

Histology is one of the most definitive methods of differentiation of CD from ITB. Features described to be only found in ITB are caseating granulomata and confluent granulomata. Large numbers of granulomata (≥10 per biopsy site), large granulomata (≥0.05 mm), ulcers lined by epithelioid histiocytes and disproportionate submucosal inflammation are other features suggested to support ITB. Acid-fast bacilli in biopsy specimens, although very specific, are not frequently encountered [2].

Polymerase chain reaction of biopsy specimens for mycobacterial DNA is rapid and reliable but still has false positives and negatives. There has been suggestion that faecal PCR is more sensitive than tissue PCR, but this needs to be investigated further [2]. Culture for *M. tuberculosis* from biopsy although definitive, is low in sensitivity (50 %) [2]. Specimens for TB culture was not taken at colonoscopy from this patient.

Although positivity of a tuberculin skin test (TST) and interferon gamma-release assay (IGRA) is supportive of ITB, in areas with a high prevalence of TB this may be an incidental positivity due to past TB exposure or in the case of TST also non-tuberculous mycobacteria or BCG vaccination. Furthermore, the use of corticosteroids and immunosuppressive drugs in a patient with IBD may limit the diagnostic utility of both these tests [2].

When all else fail, clinicians have a tendency to treat for ITB and monitor the response. This approach is safe given the potential for life threatening flare up of ITB, if treated as for CD in the setting of missed ITB, especially with immunosuppressive agents and biologics. Clinical studies which have assessed this approach have suggested that a short term (2–3 month) anti-TB treatment trial followed by re-endoscopy to look for healing may be valuable in the differential diagnosis of difficult cases [4]. Fortunately for this patient despite use of steroid, azathioprine, 6-MP and infliximab, there was no life threatening systemic flare up of tuberculosis.

## Conclusion

This case illustrates the importance of reconsidering ITB in an endemic stetting, in a patient with ‘diagnosed’ CD and ITB excluded at onset, but who fails to respond or becomes worse with immunosuppressive therapy. The reason for this may be in most instances the initial misdiagnosis of CD and missed ITB, or less likely the development of ITB in a patient with CD while on immunosuppression.

## Abbreviations

6-MP: 6-mercaptopurine
ATT: anti-TB treatment
CD: Crohn disease
IGRA: interferon gamma-release assay
ITB: intestinal TB
PCR: polymerase chain reaction
TST: tuberculin skin test
TB: tuberculosis

## References

[1] Niriella MA, De Silva AP, Dayaratne AH, Ariyasinghe MHADP, Navarathne MMN, Peiris RSK (2010). Prevalence of inflammatory bowel disease in two districts of Sri Lanka: a hospital based survey. BMC Gastroenterol..

[2] Almadi MA, Ghosh S, Aljebreen AM (2009). Differentiating intestinal tuberculosis from Crohn’s disease: a diagnostic challenge. Am J Gastroenterol.

[3] Weng MT, Wei SC, Lin CC, Tsang YM, Shun CT, Wang JY (2015). Seminar report from the 2014 Taiwan society of inflammatory bowel disease (TSIBD) spring forum (May 24th, 2014): Crohn’s disease versus intestinal tuberculosis infection. Intest Res..

[4] Park YS, Jun DW, Kim SH, Lee HH, Jo YJ, Song MH (2008). Colonoscopy evaluation after short-term anti-tuberculosis treatment in nonspecific ulcers on the ileocecal area. World J Gastroenterol.

